# Identification of Differential Responses to an Oral Glucose Tolerance Test in Healthy Adults

**DOI:** 10.1371/journal.pone.0072890

**Published:** 2013-08-22

**Authors:** Ciara Morris, Colm O’Grada, Miriam Ryan, Helen M. Roche, Michael J. Gibney, Eileen R. Gibney, Lorraine Brennan

**Affiliations:** 1 UCD Institute of Food and Health, University College Dublin, Belfield, Dublin, Rep. of Ireland; 2 UCD Conway Institute of Biomolecular and Biomedical Research, University College Dublin, Belfield, Dublin, Rep. of Ireland.; University of Tor Vergata, Italy

## Abstract

**Background:**

In recent years an individual’s ability to respond to an acute dietary challenge has emerged as a measure of their biological flexibility. Analysis of such responses has been proposed to be an indicator of health status. However, for this to be fully realised further work on differential responses to nutritional challenge is needed. This study examined whether metabolic phenotyping could identify differential responders to an oral glucose tolerance test (OGTT) and examined the phenotypic basis of the response.

**Methods and Results:**

A total of 214 individuals were recruited and underwent challenge tests in the form of an OGTT and an oral lipid tolerance test (OLTT). Detailed biochemical parameters, body composition and fitness tests were recorded. Mixed model clustering was employed to define 4 metabotypes consisting of 4 different responses to an OGTT. Cluster 1 was of particular interest, with this metabotype having the highest BMI, triacylglycerol, hsCRP, c-peptide, insulin and HOMA- IR score and lowest VO_2max_. Cluster 1 had a reduced beta cell function and a differential response to insulin and c-peptide during an OGTT. Additionally, cluster 1 displayed a differential response to the OLTT.

**Conclusions:**

This work demonstrated that there were four distinct metabolic responses to the OGTT. Classification of subjects based on their response curves revealed an “at risk” metabolic phenotype.

## Introduction

It is becoming increasingly accepted that the response to diet, is under the influence of many factors including genetic, epigenetic and metabolic factors [1]. Therefore, to move from current population based dietary guidelines into personalised nutritional advice, responses to dietary challenges need to be investigated with an emphasis on differential response. Such research could provide insight into metabolic status based on nutrient-specific responses [2]. The term metabotype has emerged in the literature and it defines a metabolic phenotype that classifies an individual in a particular category. These metabotypes can be used to define differential responses to diet, therapeutic intervention or physical challenges [3–6]. In the field of pharmacology the concept of the metabotype has gained significant momentum and the area of pharmaco-metabonomics has emerged [4,7–10]. This approach has been successfully used to define a pretreatment metabotype predictive of response to sertraline or placebo in depressed outpatients, revealing that pretreatment metabotypes may predict optimal therapy strategies [4]. More recently, application of this identified two subgroups of subjects with positive lipid response to fenofibrate therapy and with different underlying disturbances in lipoprotein metabolism [10]. In the field of human nutrition, distinct metabotypes have shown differential response to dietary interventions and the concept of using this approach to identify response to interventions has emerged [5].

In recent years, the use of meal challenges such as an oral glucose tolerance test (OGTT) and an oral lipid tolerance test (OLTT) to identify subtle changes in nutrition studies has gained momentum [11–13]. Healthy humans have the ability to maintain homeostasis through a multitude of nutritionally regulated processes. Therefore time–dependent changes in response to food is of great importance and provides information about the health of an individual. A recent study [14] revealed that plasma metabolomics and proteomics profiling after a postprandial challenge provided additional metabolic changes related to the dietary intervention not observed in non-perturbed conditions. Similarly by applying a highly controlled 4 day challenge protocol to 15 young healthy male volunteers inter-individual variation in phenotypically similar volunteers was enhanced by the challenges revealing metabotypes not observable in baseline metabolite profiles [15]. The findings from these challenge tests has revealed detailed insights into complex metabolic changes induced by OGTTs/OLTTs and offer novel perspectives on the regulation of glucose and lipid metabolism.

Development and expansion of the metabotyping approach to include responses to the standard OGTT is an important step in the use of these challenge tests in nutrition. The objective of the present work was to develop the concept of metabotyping encompassing the glucose response during an OGTT and to explore the phenotypes underlying the differential responses.

## Materials and Methods

### Ethics Statement

Ethical approval was obtained from the Research Ethics Committees in University College Dublin (UCD) (LS-08-43-Gibney-Ryan) and the study was conducted according to the principles expressed in the Declaration of Helsinki. Subjects were informed about the experimental procedures and purpose of the study prior to giving written consent.

### Subjects

This study is part of a research project under the Joint Irish Nutrigenomics Organisation which aims to create an extensive database combining information derived from three Irish Cohorts including the Metabolic Challenge Study (MECHE), the National Adult Nutrition Study (NANS) and the Trinity Ulster Department of Agriculture Study (TUDA) (www.ucd.ie/jingo/). The results from the MECHE study are presented here. Two hundred and fourteen subjects aged 18-60 years were recruited following a detailed screening session which included evaluation of the following parameters: fasting glucose, triacylglycerols, HDL, LDL and hemoglobin concentrations. Subjects were randomised to one of three groups; 76 subjects were randomized to receive an OGTT and an OLTT on two separate clinical visits, 69 subjects were randomised to receive an OGTT on two separate clinical visits and 69 subjects were randomized two OLTTs on two separate occasions.

### Sample Collection

Following a 12 hour overnight fast, second void urine and blood samples were collected. The urine was immediately centrifuged at 1800g for 10 minute at 4^0^C, and 1ml aliquots were stored at -80^0^C. All individuals underwent a 75-g OGTT according to the recommendations of the World Health Organization (WHO)/International Diabetes Federation (IDF). Venous blood samples were taken directly before (= 0 min) and during the OGTT (10, 20, 30, 60, 90, and 120 min). As detailed in the online supplemental data, the OLTT consisted of 100 mL Calogen (Nutricia, Ireland) combined with 50 mL Liquid Duocal (SHS Nutrition, Netherlands) for a total of 150 mL with a fat content of 54 grams (Table S1). Blood samples were taken directly before (= 0 min) and at 60, 120, 180, 240, and 300 min during the OLTT.

Serum and plasma samples were collected using serum tubes containing a clot activator coating, EDTA-coated evacuated tubes and tubes containing lithium heparin. The serum samples were allowed to clot for 30 minutes at room temperature. EDTA and lithium heparin tubes were placed directly on ice. All blood samples were centrifuged at 1800 x g for 15 minutes at 4^0^C and 500 ml aliquots were stored at -80^0^C until subsequent analysis.

### Anthropometric, Body Composition and Fitness tests

Height was measured using a wall-mounted stadiometer and weight was measured on a calibrated beam balance platform scale. Percentage body fat was measured via an air-displacement plethysmograph (BOD-POD GS system, Cranlea, UK), which has been shown to be an accurate method for assessing body composition in adults [16]. Percentage body fat measurements were measured in fasting state. Following consumption of a breakfast containing 558 kcal, 5 g fat, 108 g carbohydrate and 72 g protein, a measure of maximal oxygen consumption (VO_2max_) was carried out on an electronically braked cycle ergometer (Ergoline 500, Bosch, Germany). To individualise the increment of exercise intensity during the VO_2max_ test, the workload of each step was calculated from the theoretical VO_2max_. Consequently subjects underwent a test with the same relative incremental workload. The test consisted of a three minute warm up at baseline followed by four minute steady state workloads at 15%, 35%, 55% and 75%. Recovery to baseline (VO_2_ and heart rate returned to baseline value and an R value under 1 was achieved) was recorded after each increment. The subjects performed the test on an electronically braked cycle ergometer (Ergoline Bosh 500). Heart rate was monitored continuously throughout the test and metabolic and ventilatory responses were assessed using a computer based breath to breath exercise analysing system (Cosmed Quark B2, H Evans, Ireland).

### Biochemical and Immune Parameters

Clinical chemistry analysis was performed using a RxDaytona™ chemical analyser autoanalyser (Randox Laboratories, Crumlin, UK) and Randox reagents. Details of the analytes and methods are as follows: total cholesterol (cholesterol oxidase), HDL-cholesterol (direct clearance), glucose (glucose oxidase), triacylglycerol (lipase/glycerol kinase colorimetric), C reactive protein (immunoturbidimetric) and NEFA (lipase/glycerol kinase colorimetric).

The Evidence Investigator™ (Randox Laboratories, Crumlin, Northern Ireland) metabolic array I kit was used for the simultaneous measurement of C-peptide, Insulin, Resistin, and Tumor Necrosis Factor-α. Standard quality control procedures were followed on both analysers to ensure the integrity of the data.

HOMA-IR score was calculated using the formula: (Fasting insulin µU/mL x fasting glucose mmol/L)/ 22.5 and using: (Fasting insulin pmol/l x fasting glucose mmol/L)/ 22.5.

### Clustering and Statistical Analysis

Clusters were determined using mix tools analysis in R as described previously by Benaglia and colleagues [17]. Biochemical data, body composition data and fitness data were compared between clusters using general liner model (GLM) analysis. Data is presented as mean ± standard error of mean (SEM). Statistical analysis was performed using the statistical package PASW for windows V.18.0.0 (SPSS, Chicago, USA). Analysis of variance (ANOVA) was used to examine mean differences in continuous data. 

## Results

### Identification of four distinct metabotypes based on response to an OGTT

**Figure 1:**
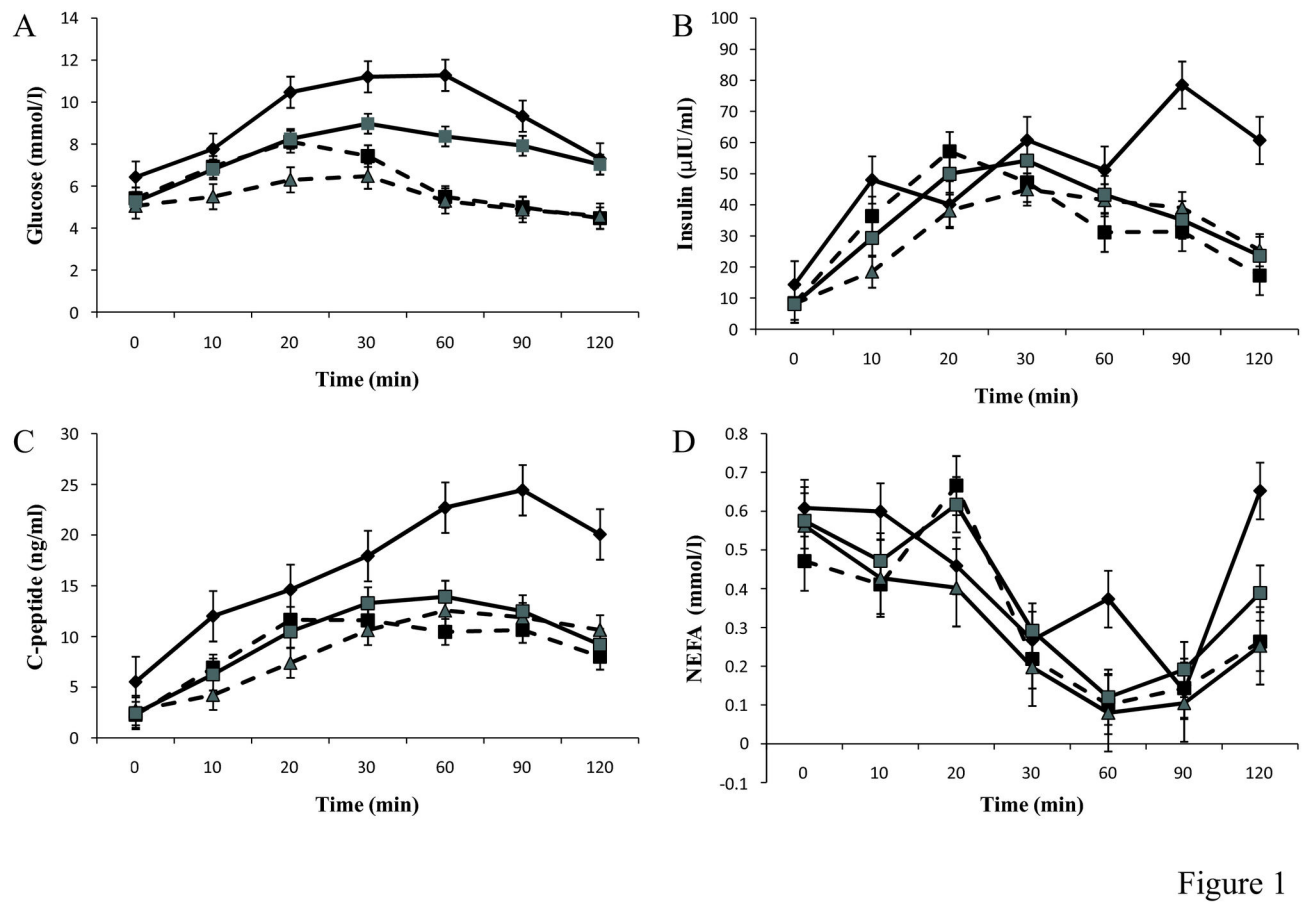
Identification of four distinct metabolic responses. (A) Glucose concentration during the oral glucose tolerance test (OGTT) across the four cluster groups (B) Insulin concentration during an OGTT across the four cluster groups (C) C-peptide concentration during an OGTT across the four cluster groups. (D) NEFA concentration during an OGTT across the four cluster groups. Cluster 1 is represented by a black line and ♦ marker. Cluster 2 is represented by black dashed line and ■ marker. Cluster 3 is represented by a grey dashed line and grey ▲ marker. Cluster 4 is represented by a grey line and a grey ■ marker. All values are mean ± SEM. The AUC was significantly different across the 4 cluster for glucose (*p*= 1.6 x 10^-16^), insulin (*p*= 1.5 x 10^-3^) and C-peptide (*p*= 2.6 x 10^-6^).

For the present study, subjects (n=145) who underwent an OGTT were selected and from these only subjects with a complete dataset for glucose curves were used (n=116). The overall characteristics of the subjects are presented in Table 1. Metabotypes were identified by analysis of the response to an OGTT using mix tools analysis in R as described previously [17]. Analysis revealed four distinct metabotypes (Figure 1A). Cluster 1 was characterised by the highest BMI, highest percentage body fat and lowest VO_2max_. Cluster 2 had the highest mean VO_2max_ while cluster 3 had the lowest BMI and lowest percentage body fat (Table 2). There was no significant difference in the distribution of gender across the clusters.

**Table 1:** Demographics of study population.

	**Mean ± S.E.M**
**Age (years)**	32.2 ± 0.74
**Weight (Kg)**	75.1 ± 1.1
**BMI (kg.m^-2^)**	24.8 ± 0.3
**Body fat (%)**	26.1 ± 0.8
**VO_2MAX_ (ml/min/kg)**	42.1 ± 1.2
**Glucose 0 (mmol/l)**	5.4 ± 0.1
**HOMA-IR**	2.19 ± 0.16

Data are means ± SEM.

**Table 2:** Demographics of the cluster groups.

	**Cluster 1 (n=17)**	**Cluster 2 (n=34)**	**Cluster 3 (n=20)**	**Cluster 4 (n=45)**	**P Value**
**Gender (M/F)**	10/7	21/13	7/13	19/26	ns
**Age (years)**	38 ± 3^3^	33 ± 2	29 ± 2^1^	31 ± 1	4.1 x 10^-2^
**Weight (Kg)**	87.3 ±5.7^2,3,4^	74.9 ± 2.8^1^	68.0 ± 2.4^1^	73.9 ± 2.2^1^	5.0 x 10^-3^
**BMI (kg.m^-2^)**	29.4 ± 2.1^2,3,4^	24.5 ± 0.8 ^1^	22.7 ± 0.5 ^1^	24.4 ± 0.5 ^1^	3.2 x 10^-4^
**Waist Circumference (cm)**	95.7 ± 4.9^2,3,4^	83.2 ± 2.4^1^	79.4 ± 1.5^1^	83.4 ± 1.6^1^	2.0 x 10^-3^
**Body fat (%)**	30.9 ± 6.5	23.5 ± 1.4	24.5 ± 3.0	26.9 ± 1.6	ns
**VO_2MAX_ (ml/min/kg)**	36 ± 3^2^	47 ± 2^1^	40 ± 1	41 ± 2	1.7 x 10^-2^

Data are means ± SEM. P value determined using univariant general linear model with Bonferroni posthoc analysis for group comparison. The numbers indicate which cluster groups are significantly different (P< 0.05). ns = non-significant

With regards the glucose curves, Cluster 1 had the highest baseline glucose, the highest peak glucose value and the highest glucose at 120 minutes (Table 3). Cluster 2, 3 and 4 had similar baseline glucose values (Table 3). Cluster 2 had the earliest peak for glucose with a peak value achieved at 20 minutes. Cluster 3 displayed the lowest response with peak glucose values reaching only 6.47 mmol/l compared to the high value of 11.28 mmol/l for cluster 1 (Figure 1). Cluster 1 and cluster 4 displayed the highest peak values and neither returned to baseline values at 120 minutes. Whereas the glucose values for cluster 2 and 3 returned to values lower than the baseline values at 120 minutes.

**Table 3:** Glucose response during oral glucose tolerance test for the four metabotypes.

	**Cluster 1 (n=17)**	**Cluster 2 (n=34)**	**Cluster 3 (n=20)**	**Cluster 4 (n=45)**	**P Value**
**Glucose 0 (mmol/l)**	6.4 ± 0.2^2,3,4^	5.42 ± 0.1^1^	5.05 ± 0.2^1^	5.26 ± 0.1^1,3^	5.6 x 10^-11^
**Glucose 10 (mmol/l)**	7.76 ± 0.38^2,3,4^	6.92 ± 0.13^1,3^	5.50 ± 0.23^1,2,4^	6.79 ± 0.10^1,3^	7.2 x 10^-14^
**Glucose 20 (mmol/l)**	10.47 ± 1.16^2,3,4^	8.11 ± 0.19^1,3^	6.30 ± 0.24^1,2,4^	8.25 ± 0.13^1,3^	1.5 x 10^-26^
**Glucose 30 (mmol/l)**	11.20 ± 0.53^2,3,4^	7.43 ± 0.23^1,3,4^	6.47 ± 0.24^1,2,4^	8.97 ± 0.16^1,2,3^	5.2 x 10^-30^
**Glucose 60 (mmol/l)**	11.28 ± 0.66^2,3,4^	5.49 ± 0.29^1,4^	5.29 ± 0.31^1,4^	8.37 ± 0.23^1,2,3^	1.2 x 10^-42^
**Glucose 90 (mmol/l)**	9.33 ± 0.30^2,3,4^	4.99 ± 0.26^1,4^	4.88 ± 0.28^1,4^	7.92 ± 0.24^1,2,3^	5.3 x 10^-38^
**Glucose 120 (mmol/l)**	7.30 ± 0.7^2,3^	4.47 ± 0.22^1,4^	4.57 ± 0.28^1,4^	7.02 ± 0.21^2,3^	4.0 x 10^-30^

Data are means ± SEM. P value determined using univariant general linear model with Bonferroni posthoc analysis for group comparison. The letters indicate which cluster groups are significantly different (P< 0.05).

### Characterisation of the metabotypes

Cluster 1 was characterised by having the highest HOMA-IR score, and the highest triacylglycerol, hsCRP, c-peptide and insulin concentrations. Cluster 3 was characterised by the lowest, triacylglycerol, hsCRP, insulin and HOMA- IR score (Table 4). Over the course of the OGTT the dynamic response of insulin and C-peptide concentrations were also measured. Interrogation of these response curves revealed that the insulin response curves were similar for cluster 2, 3 and 4. However, cluster 1 displayed a significantly different insulin response curve with a significantly larger area under the curve (AUC) compared to the other 3 groups. Additionally peak insulin was achieved at the much later timepoint of 90 minutes and insulin values at 120 minutes were not restored to baseline values (Table 5, Figure 1B). Similarly the AUC for the C-peptide response in cluster 1 was significantly higher (p = 2.6 x 10^-6^) than the other clusters (Figure 1C). Moreover, the c-peptide values for cluster 1 at 120 minutes were significantly higher than baseline values.

**Table 4:** Metabolic characteristics of the four metabotypes.

	**Cluster 1 (n=17)**	**Cluster 2 (n=34)**	**Cluster 3 (n=20)**	**Cluster 4 (n=45)**	**P Value**
**INS (µIU/ml)**	15.17 ± 4.47^2,3,4^	9.12 ± 1.25 ^1^	7.90 ± 2.03 ^1^	8.09 ± 0.8 ^1^	1.1 x 10^-4^
**C-peptide (ng/ml)**	5.52 ± 1.62^2,4^	2.54 ± 0.37^1^	2.71 ± 1.09	2.46 ± 0.28^1^	1.3 x 10^-2^
**Cholesterol (mmol/l)**	4.79 ± 0.29^3^	4.47 ± 0.14	3.96 ± 0.19^1^	4.48 ± 0.13	4.6 x 10^-2^
**HDL Cholesterol (mmol/l)**	1.19 ± 0.22	1.35 ± 0.05	1.40 ± 0.08	1.40 ± 0.04	ns
**Triacylglycerols (mmol/l)**	1.79 ± 0.31^2,3,4^	0.96 ± 0.08^1^	0.77 ± 0.06 ^1^	0.94 ± 0.05 ^1^	2.0 x 10^-6^
**NEFA (mmol/l)**	0.65 ± 0.1	0.49 ± 0.1	0.51 ± 0.06	0.59 ± 0.07	ns
**hsCRP (mmol/l)**	3.1 ± 0.8^3^	1.7 ± 0.4	0.9 ± 0.3^1^	1.5 ± 0.2	1.8 x 10^-2^
**Resistin (ng/ml)**	4.67 ± 0.6	4.01 ± 0.21	4.46 ± 0.41	4.42 ± 0.27	ns
**TNFα (pg/ml)**	5.70 ± 0.83	5.11 ± 0.67	5.30 ± 0.77	5.02 ± 0.43	ns
**HOMA-IR**	2.71 ± 0.48^2,3,4^	1.95 ± 0.29^1^	1.24 ± 0.16 ^1^	1.70 ± 0.2 ^1^	1.2 x 10^-3^

Data are means ± SEM. P value determined using univariant general linear model with Bonferroni posthoc analysis for group comparison. The letters indicate which cluster groups are significantly different (P< 0.05).

**Table 5:** Area under the curve during oral glucose tolerance test and oral lipid tolerance test.

	**Cluster 1 (n=17)**	**Cluster 2 (n=34)**	**Cluster 3 (n=20)**	**Cluster 4 (n=45)**	**P Value**
**Glucose_AUC_ OGTT**	963.2 ± 60.0^2,3,4^	629.8 ± 10.8^1,3^	607.6 ± 28.6^1,4^	747.3 ± 9.5^1,2,3^	1.6 x 10^-16^
**Insulin_AUC_ OGTT**	7242.1 ± 1237.4^2,3,4^	4064.4 ± 413.6^1,4^	4236.5 ± 442.3^1^	4607.8 ± 310.9^1^	1.5 x 10^-3^
**C-peptide_AUC_ OGTT**	2368.1 ± 345.1^2,3,4^	1175.9 ± 92.2^1^	1212.0 ± 109.7^1^	1368.5 ± 91.0^1^	2.6 x 10^-6^
**NEFA_AUC_ OGTT**	44.06 ± 6.8.9	28.79 ± 1.56	23.84 ± 4.25	34.93 ± 1.69	ns
**Glucose_AUC_ OLTT**	1670.9 ± 79.5^2,3,4^	1464.9 ± 87.4^1^	1385.9 ± 86.1^1^	1508.1 ± 87.9^1^	7.1 x 10^-4^
**Insulin_AUC_ OLTT**	9794 ± 1543.3^2,3,4^	3881.2 ± 563.2^2,3,4^	5423.6 ± 565.2^1,2,4^	945.3 ± 52.3^1,2,3^	1.7 x 10^-3^
**C-peptide_AUC_ OLTT**	3598.4 ± 569.3^2,3,4^	1171.5 ± 88.3^1^	1483 ± 78.4^1^	972.4 ± 68.3^1^	2.3 x 10^-5^
**Triacylglycerol_AUC_ OLTT**	240.92 ± 15.95 ^2,3,4^	156.03 ± 15.54^1^	127.50 ± 13.15^1^	139.60 ± 12.48^1^	9.4 x 10^-4^
**NEFA_AUC_ OLTT**	589.14 ± 53.59	372.65 ± 49.79	311.70 ± 90.75	375.10 ± 38.77	1.7 x 10^-4^

Data are means ± SEM. P value determined using univariant general linear model with Bonferroni posthoc analysis for group comparison. The letters indicate which cluster groups are significantly different (P< 0.05).

**Figure 2:**
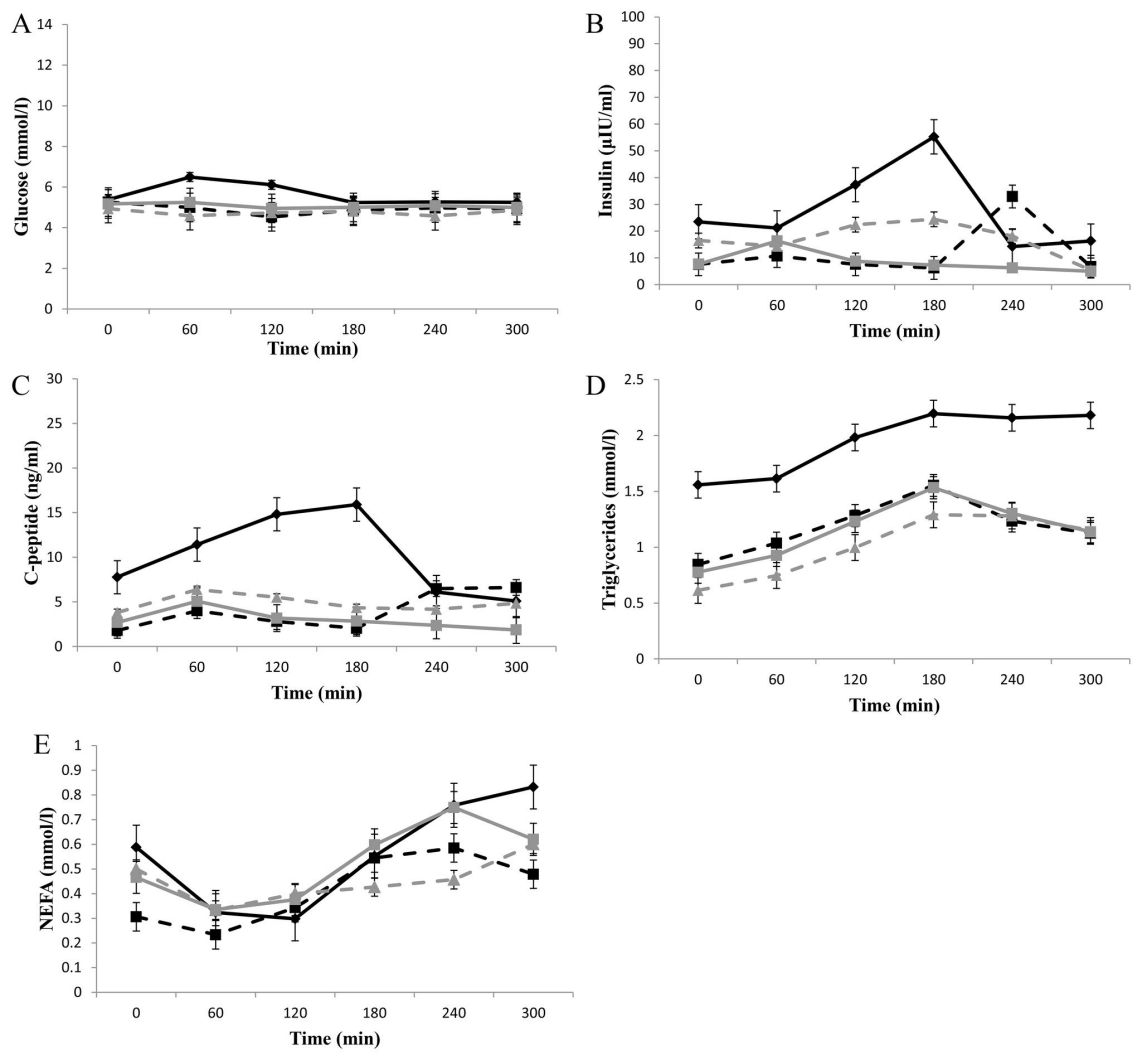
Differential responses to an OLTT. (A) Glucose concentration during the OLTT test across the four cluster groups (B) Insulin concentration during the OLTT across the four cluster groups (C) C-peptide concentration during the OLTT across the four cluster groups. (D) Triacylglycerol concentration during the OLTT across the four cluster groups. (E) NEFA concentration during oral lipid tolerance test across the four cluster groups. Cluster 1 is represented by a black line and ♦ marker. Cluster 2 is represented by black dashed line and ■ marker. Cluster 3 is represented by a grey dashed line and ▲ marker. Cluster 4 is represented by a grey line and a ■ marker. All values are mean ± SEM. The AUC was significantly different across the 4 cluster for glucose (*p*= 7.1 x 10^-4^), insulin (*p*= 1.7 x 10^-3^), C-peptide (*p*= 2.3 x 10^-5^), triacylglycerols (*p*= 9.4 x 10^-4^), and NEFA (*p*= 1.7 x 10^-4^).

Interrogation of the glucose, insulin, C-peptide and triacylglycerol response during an OLTT revealed that cluster 1 had a significantly higher AUC (p = 7.1 x 10^-4^) during the glucose response to the OLTT, with a peak glucose response at 60 minutes compared to the other clusters where the glucose values remained constant (Figure 2A). The insulin response to the OLTT was significantly different across the clusters with the highest AUC for cluster 1 (*p*= 1.7 x 10^-3^). Cluster 2 displayed a late insulin response with peak values at 240 minutes. With respect to the C-peptide response to the OLTT cluster 1 had a significantly higher AUC (p = 2.3 x 10^-5^) with a peak value at 180 minutes. The TAG response to the OLTT was significantly higher in cluster 1 (p= 1.7 x 10^-4^) (Table 5).

### Estimates of insulin resistance and β-cell function across the four metabotypes

**Figure 3:**
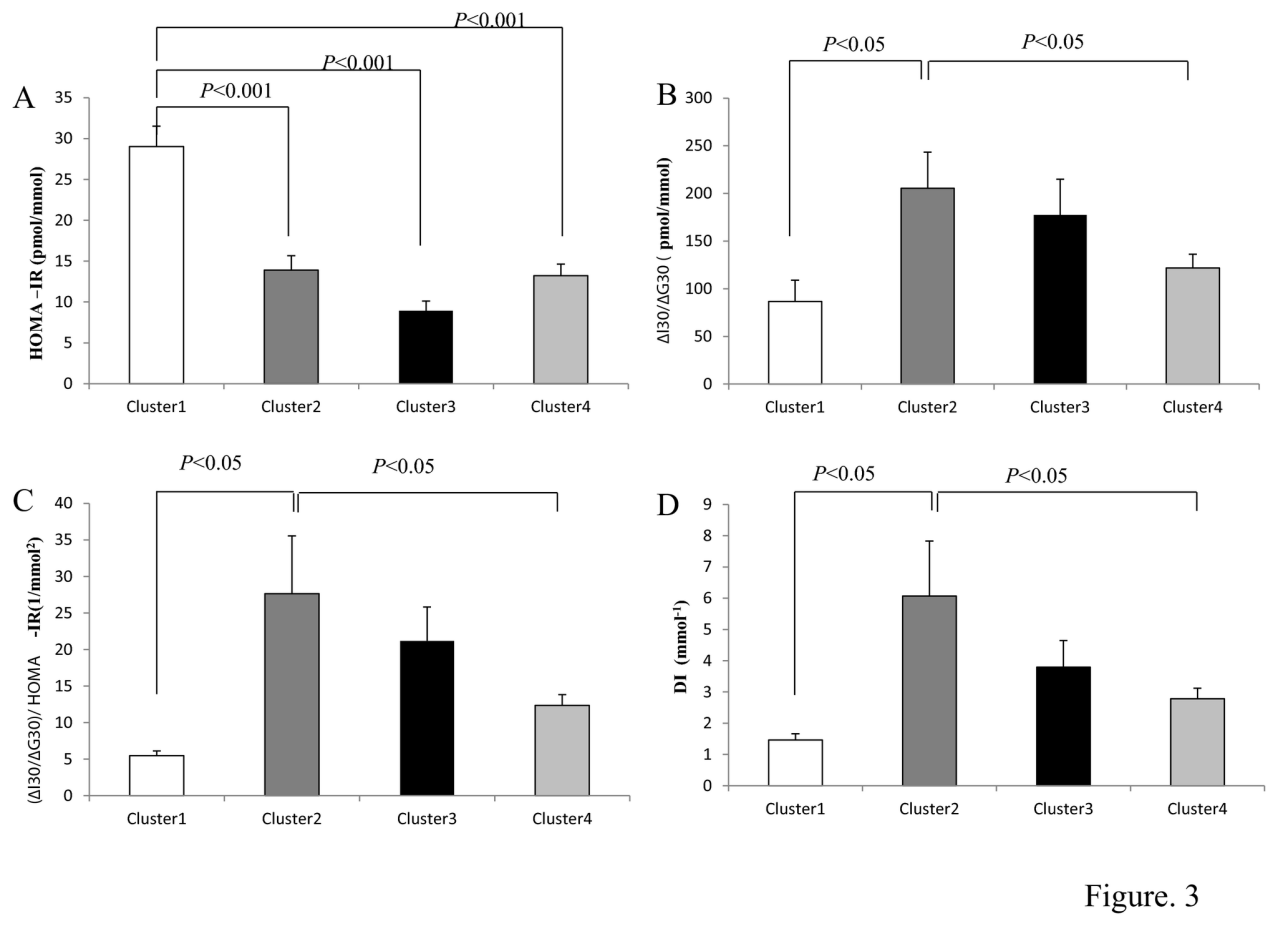
Insulin resistance and β-cell function across the four metabotypes. (A) Insulin resistance determined by the HOMA-IR (B) β-cell function quantified as ∆I30/∆G30 (C) (∆I30/∆G30)/HOMA-IR and (D) Disposition Index determined as (∆I30/∆G30) x 1/ fasting insulin from an OGTT in 116 individuals. Cluster 1- White bar (*n*=17), Cluster 2 – Dark grey bar (*n* =34), Cluster 3 – Black bar (*n* = 20) and Cluster 4 – Light grey bar (*n* =45). As glucose tolerance declined, insulin resistance increased and β-cell function deteriorated. HOMA-IR for cluster 1 was significantly different to cluster 2 (*p*<0.001), cluster 3 (*p*<0.001) and cluster 4 (*p*<0.001). Cluster 1 β-cell function quantified as ∆I30/∆G30 was significantly different to cluster 2 (*p*<0.05) and Cluster 1 β-cell function normalised to HOMA-IR quantified as (∆I30/∆G30)/HOMA-IR was significantly different to cluster 2 (*p*<0.05). HOMA-IR was calculated as follows: Fasting insulin pmol/l x fasting glucose mmol/L)/ 22.5.

β-cell function was assessed as the ratio of the incremental insulin to glucose responses over the first 30 minutes during the OGTT [18] (ΔI_30_/ΔG_30_). Insulin resistance is known to be a critical modulator of the insulin response to a stimulus, with insulin resistance increasing insulin release [19]. Thus, we also adjusted ΔI_30_/ΔG_30_ for the degree of insulin resistance because this varied across cluster groups (Figure 3B). Dividing ΔI_30_/ΔG_30_ by the HOMA-IR gave an adjusted measure of β-cell function (ΔI_30_/ΔG_30_/HOMA-IR) that accounted for variation in insulin resistance (Figure 3C). Application of this measure across the cluster groups revealed that cluster 1 had the lowest β-cell function and that cluster 2 had the highest. In addition, the oral disposition index (DI) was assessed as previously described [20]. This provides a measure of β-cell function adjusted for insulin sensitivity and is predictive of diabetes. As with the other measures of β-cell function the DI was lowest for cluster 1.

## Discussion

In recent years the use of the response to a challenge of homeostasis has become an important tool in nutrition research, as challenging the individual is much more informative than static homeostatic measures [21–23]. The present work extended this concept by developing a metabolic phenotyping approach based on the glucose response to a standard OGTT. This phenotyping approach identified 4 metabotypes consisting of 4 different responses to an OGTT. Cluster 1 was of particular interest, with this metabotype having the highest BMI, triacylglycerol, hsCRP, c-peptide, insulin and HOMA- IR score and lowest VO_2max_. level. Cluster 1 also had a reduced beta cell function and a differential response to insulin and c-peptide during an OGTT. Furthermore even though not modeled, cluster 1 displayed a differential response to the OLTT. Overall, this approach successfully identified metabolic phenotypes and could be used to identify at risk phenotypes or metabolically disturbed phenotypes.

The phenotyping approach used here is based on modeling the response curves and previous data has shown that there is biological meaning in the shape of the curve during a challenge test. Tschritter and colleagues [24] confirmed that the plasma glucose shape during an OGTT was dependent on both glucose tolerance and gender with genetic factors also playing a role. The concept of a “shape” index based on the extent and the direction of the plasma glucose change in the second hour during an OGTT emerged from this work. Application of this, lead the authors to conclude that the “shape” index may be a useful metabolic screening parameter in epidemiological and genetic association studies. Further studies have also shown the biological importance of the shape of the curve during an OGTT. Tura and colleagues [25] analysed the shape of the glucose, insulin, and c-peptide curves during a 3-h OGTT and reported that the majority of the glucose curves were monophasic and that although complex shapes were less frequent they were not rare. Furthermore there was a tendency towards the amelioration of the metabolic condition with increasing complexity of the shape, as indicated by lower glucose concentrations, improved insulin sensitivity and β-cell function. More recently, Krishnan and colleagues assessed the differential response to a low and high glycemic meal and identified 3 response groups [26]. Although some of these groups were small (n=3) it adds weight to the method developed in this paper and strengthens the proposed utility of this metabotype approach.

Cluster 1 is of particular interest and displayed a very different response for glucose, insulin and c-peptide during the glucose challenge compared to the other 3 metabotypes. To probe the basis for this response we estimated the β-cell function and insulin resistance [18] of each of the 4 clusters. Cluster 1 had the lowest β-cell function, suggesting that β-cell dysfunction, is present in this phenotype and may be the underlying driver of the response curves. Identification of subjects with sub-functional β-cells is of clinical relevance considering that previous work has suggested that β-cell function is a critical component in the pathogenesis of type 2 diabetes [27–30]. Additionally the oral disposition index was lowest in this group which has been demonstrated to be predictive of development of diabetes over 10 years [20].

Cluster 1 had higher concentrations of triacylglycerols compared to the other three clusters and interestingly cluster 1 also displayed a differential response to the OLTT. Our results are in agreement with the work of Harano et al. [31] and Wybranksa et al. [32], which pointed to a strong correlation between insulin output following both a glucose challenge and a high-fat challenge. However, in contrast to these studies the carbohydrate content was minimal in the present study and this response to glucose and insulin after the lipid challenge could be defined to one cluster group/metabotype. The elevated response in cluster 1 indicated an inherent metabolic dysfunction in cluster 1 and demonstrates that the metabotyping approach could be used to define individuals at risk.

The raised baseline and response concentrations of the triacylglycerols in cluster 1 is interesting in the context of β-cell dysfunction: cluster 1 also had the lowest β-cell function. In recent years a number of papers have clearly demonstrated that raised concentrations of free fatty acids results in β-cell toxicity by altering gene expression, function, survival and growth [33–40]. From this work the term lipotoxicity has emerged. Elevated free fatty acids in the presence of high glucose concentrations results in glucolipotoxicity and a number of mechanisms have been proposed to explain the emergence of glucolipotoxic conditions including oxidative stress, ER stress, cytokine induced apoptosis and hypoxia. Although NEFA concentrations were not significantly different across the clusters, cluster 1 had higher triacylglycerol concentrations and triacylglycerols are a key factor for the development of impaired glucose-stimulated insulin secretion (GSIS) [41,42]. Thus the increased concentrations of triacylglycerols in cluster 1 may result in a lipotoxic environment and impaired GSIS in the β-cells and therefore contribute to the decreased insulin sensitivity of the subjects within this cluster.

In conclusion, we have developed the concept of metabotyping encompassing the glucose response during an OGTT and demonstrate clearly that we can define distinct metabolic groups within a study population. Such an approach clearly identified an at risk phenotype within the group. Tailoring lifestyle and dietary advice to these metabotype groups is the next step in promoting the use of such an approach.

## Supporting Information

Table S1 **Nutritional Composition of Metabolic Challenges.**
(DOCX)
