# Supplementary material for: Identification of Differential Responses to an Oral Glucose Tolerance Test in Healthy Adults
Source: PLoS One. 2013 Aug 22;8(8):e72890. doi: 10.1371/journal.pone.0072890 (PMC3749984; doi:10.1371/journal.pone.0072890)
Supplement: Table S1 — Nutritional Composition of Metabolic Challenges. (DOCX) [file pone.0072890.s001.docx]

**Online Supplemental Material**

**Table S1** Nutritional Composition of Metabolic Challenges

|  | **OGTT** | **OLTT** |
| --- | --- | --- |
| **Calories (kcal)** | 300 | 533 |
| **Protein (g)** | 0 | 0 |
| **Carbohydrate (g)** | 77 | 11.85 |
| **Of which sugars (g)** | 75 | 1.05 |
| **Total Fat (g)** | 0 | 53.95 |
| **SFA (g)** | 0 | 7.05 |
| **MUFA (g)** | 0 | 31.10 |
| **PUFA (g)** | 0 | 15.75 |
| **Sodium (mmol)** | 0 | 0.61 |

The OGTT consisted of 75g glucose (anhydrous) in 150 mL of water. The OLTT consisted of 100 mL Calogen (Nutricia, Ireland) combined with 50 mL Liquid Duocal (SHS Nutrition, Netherlands) for a total of 150 mL with a fat content of 54 grams.
